# The Effects of Phosphate Compounds on the Microstructure and Mechanical Properties of Fly Ash Geopolymer Mortars

**DOI:** 10.3390/ma17225451

**Published:** 2024-11-08

**Authors:** Piotr Prochon, Tomasz Piotrowski, Maja Kępniak

**Affiliations:** Department of Building Materials Engineering, Faculty of Civil Engineering, Warsaw University of Technology, Armii Ludowej 16, 00-637 Warsaw, Poland; tomasz.piotrowski@pw.edu.pl (T.P.); maja.kepniak@pw.edu.pl (M.K.)

**Keywords:** AAMs, geopolymers, mortars, biomass fly ash, microstructure, mechanical properties, phosphate compound

## Abstract

Coal-fired power plants are a main source of energy in Poland. In the rapidly growing demand for the reduction of CO_2_ emission in the energy industry, the use of biomass for energy purposes has increased significantly. The combustion of biomass results in the generation of fly ash, with higher levels of CaO, K_2_O, P_2_O_5_, in contrast to the fly ash derived from the combustion of coal. The aim of this study was to examine the influence of phosphate compounds on fly ash-based geopolymer mortars. Geopolymers were made by mixing two types of fly ash—one from the combustion of wood biomass and the second from the combustion of coal in a heat and power station. Basic activators (NaOH and Na_2_SiO_3_) were used for the alkali activation. The maximum level of tetraphosphorus decaoxide addition was established at 5% of the total mass of the aluminosilicate precursors mass. The results showed that the phosphate oxide concentration within the specimens demonstrated a positive correlation with flexural and compressive strength across all temporal intervals (7, 28, and 56 days). The porosity, however, for samples with a 5% addition of P_4_O_10_, increased more than twofold in comparison to reference samples (from 4.26% to 9.98%).

## 1. Introduction

Coal-fired power plants account for almost 80% of Poland’s total energy generation, making it one of the highest coal-dependent countries in Europe [1]. The native bituminous coal contributes nearly fifty percent to Poland’s total energy supply, highlighting its essential function within the nation’s energy composition [2]. In light of the heightened call for CO_2_ emission reductions in the energy sector, biomass energy generation has seen a notable uptick in Poland. This phenomenon is not solely confined to the combustion of conventional woody materials, but extends to encompass various forms of biomass. The combustion of biomass results in the generation of two principal categories of waste as follows:

- Bottom ash, characterized by a coarse fraction (predominantly quartz), unburned particles, and mineral impurities derived from the biomass;
- Fly ash, which is extracted from the exhaust gas stream outside the combustion chamber, comprising the finest particles and primarily inorganic components [3,4].

The quantity and quality of biomass fly ashes exhibit substantial variability contingent upon the properties of the biomass employed (species of plants, types of plant parts, growth processes, and cultivation conditions) and the combustion technology utilized (type of furnace, fuel preparation methods, and combustion temperatures) [5,6]. These components might either raise or lessen the amount of principal oxides (CaO, K_2_O, P_2_O_5_, SiO_2_, and Al_2_O_3_) or trace elements (Zn, Cu, and Mn) present in the chemical framework of biomass fly ash [7,8], the levels of principal oxides, and type of raw material used highly influence the geopolymerization process [9]. Owing to the heterogeneous chemical composition, it is imperative that combustion byproducts, particularly those resulting from the co-firing of coal and biomass or solely from biomass, undergo a thorough assay for their individual oxide components to facilitate their utilization as precursors for alkali activation.

To this point, empirical evidence has substantiated that the phenomenon of polymeric aluminosilicate binding is influenced by the chemical composition of the geopolymerization precursors, the mass ratio of SiO_2_ to Al_2_O_3_, the concentration of the alkali activator, as well as the conditions of curing temperature and duration [10,11,12,13,14,15,16,17,18,19]. For optimal geopolymerization, it is advised by researchers to maintain the curing temperature within the range of 60 °C to 90 °C [10,11,12,19]. Modifications to the curing temperature and the concentration of alkali activators result in a reorganization of the structural characteristics of the geopolymerization products, which is manifested through alterations in the Si/Al ratio and variations in the vibrational ranges of Si-O-Si and Al-O-Si bonds [16]. Furthermore, it has been noted that a heightened alkaline reaction (characterized by a low SiO_2_/K, Na_2_O ratio) accelerates the dissolution of Si and Al phases, enhances the formation of aluminosilicate gel, and diminishes the mechanical properties of the resulting geopolymers [11]. An increased concentration of Na_2_O, along with a SiO_2_/Na_2_O ratio of 1.4–1.6, is associated with a reduction in porosity and facilitates improved packing of geopolymer binder grains [12,13]. Concurrently, an investigation into the phase composition and microstructure conducted by alternative scholars has elucidated that irrespective of the precursor employed, the concentrations of SiO_2_ and CaO significantly influence mechanical strength, while the presence of Al_2_O_3_ enhances the formation ratio of amorphous products [14]. Given the structural characteristics of polysilane macromolecules, which are predominantly composed of SiO_4_ and AlO_4_ copolymers, alongside the presence of primary chemical constituents in alkali-activated materials (namely SiO_2_, Al_2_O_3_, CaO, and Fe_2_O_3_), a significant portion of scholarly investigations has been directed towards examining the effects of aluminum, silica, calcium, and iron compounds on the geopolymerization process [18,19,20].

Despite the extensive body of research on the chemistry and microstructural characteristics of geopolymers, certain elements of their synthesis mechanisms continue to elude comprehensive understanding. The dynamics of concurrent reactions, along with the influence of impurities present in the chemical makeup of geopolymerization precursors (like P_2_O_5_), have not been exhaustively investigated. To date, there exists a notable absence of research focused on the implications of phosphorus oxide with respect to the microstructural integrity, binding mechanisms, and mechanical attributes of geopolymers, with P_2_O_5_ content varying from 0.1% to approximately 41%, contingent upon the specific combustion byproducts employed [21]. Phosphorus compounds are predominantly referenced in the context of geopolymer synthesis utilizing orthophosphoric acid [22,23], or in scenarios where fossil waste is utilized as substitutes for geopolymerization precursors [24]. It is pertinent to note that current investigations are underway that emphasize the impact of phosphorus compounds on the hydration processes within cementitious binders [25,26,27,28,29].

In the present investigation, the influence of phosphorus oxide addition on the characteristics of alkali-activated materials is analyzed. A combination of two distinct fly ashes was employed as precursors for the alkali activation process utilizing sodium hydroxide and sodium silicate: silica fly ash resulting from coal combustion (RFA) and fly ash obtained from the combustion of wooden biomass (BFA). All the fly ashes underwent thorough examination utilizing X-ray fluorescence analysis (XRF), X-ray diffraction analysis (XRD), and scanning electron microscopy (SEM) image analysis complemented by an energy dispersive X-ray spectrometer (EDS) attachment, in addition to measurements of density, loss on ignition, and particle size distribution.

To examine the influence of phosphorus oxide on the properties of geopolymer mortars, three distinct concentrations of the phosphate compound were selected: 1%, 3%, and 5%. For the resulting mortars, analysis was performed utilizing SEM/EDS, micro-computed tomography, and Fourier Transform Infrared Spectroscopy. Gaining insight into the effects of phosphorus oxide on geopolymer mortars holds significant potential for elucidating their role as a catalyst or inhibitor in the geopolymerization process.

## 2. Materials and Methods

### 2.1. Fly Ashes

Two fly ashes were used as precursors for alkali active activation. The first sample originated from bituminous coal firing (RFA). The second sample was sourced from wooden biomass firing (BFA). Both samples were procured from the same Polish Heat and Power Station Siekierki, Warsaw, Poland.

#### 2.1.1. Density

The true density of fly ashes was quantified using a gas pycnometer. This device functions by measuring the pressure variation caused by the gas displacement, in this case helium displacement, due to a solid element within two chambers: the sample chamber and the reference chamber.

A pulverized fly ash sample, with an unidentified volume (V_m_) and identified mass (m_m_), was introduced into a sealed sample chamber of identified volume (V_ch_). Following sealing, the pressure in the sample chamber was recorded (P_ch_). The reference chamber, also of known volume (V_r_), was pressurized beyond P_ch_ (P_r_). By opening the valve separating the chambers, the system pressure (P_sys_) was equilibrated. The procedure was conducted thrice for a single mortar. The sample volume was ascertained according to the gas law, utilizing the average findings as follows:

(1) $$V_{m}=\frac{\left(P_{sys}\times V_{ch}+P_{sys}\times V_{r}-P_{ch}\times V_{ch}-P_{r}\times V_{r}\right)}{\left(P_{sys}-P_{ch}\right)},\;[{dm}^{3}]$$

where:

- V_m_—volume of pulverized sample, dm^3^;
- V_ch_—volume of sealed chamber, dm^3^;
- P_ch_—the pressure within the sample chamber, Pa;
- V_r_—reference chamber of known volume, dm^3^;
- P_r_—charged pressure, Pa;
- P_sys_—system pressure, Pa.

The density of fly ash samples was calculated from the density definition as follows:

(2) $$\rho =\frac{m_{m}}{V_{m}},\;[\frac{g}{{dm}^{3}}]$$

where:

- m_m_—mass of pulverized sample, g.

#### 2.1.2. XRF and XRD

The compositional analysis of the oxide was determined utilizing X-ray fluorescence testing (XRF, Thermo Fisher Scientific, Waltham, MA, USA). The specimens designated for XRF analysis were exposed to a fusion process involving lithium borate at temperatures surpassing 1000 °C, subsequent to which the resulting beads were subjected to examination through X-ray fluorescence spectrometry.

The mineralogical composition and amorphous content of the fly ashes were analyzed by means of Bragg-Brentano X-ray diffraction (XRD) on a Bruker-AXS D8 DAVINCI diffractometer equipped with a copper anode lamp. Diffractograms were recorded over an angular range of 5 to 120° 2θ (Cu Kα), with a measurement step of 0.01° and a measurement time of 2 s/step. The optical system of the diffractometer comprises a divergence slit with a width of 0.3° and a counter-scattering slit with a width of 2.94°. The identification of phases was conducted by comparing the recorded diffractograms with standards sourced from the COD database. The preparation of the samples for testing involved grinding the material and adding an external standard (CaF_2_) in the amount of 15% by weight. Pressed preparations were then made from the prepared material. The quantitative analysis was performed using the Rietveld method, whereby the theoretical diffractogram calculated according to the models of the detected crystal structures was matched with the experimental diffractogram.

#### 2.1.3. Loss on Ignition (LOI)

The loss on ignition (LOI) of fly ashes was assessed gravimetrically in accordance with the standards delineated in EN 196-2 [30] and EN 450-1 [31]. A sample of 1 ± 0.05 g of fly ash was placed into a crucible (m_1_). The covered crucible was subsequently positioned within a furnace maintained at a temperature of 950 ± 25 °C. Following a heating duration of 5 min, the lid of the crucible was removed and the crucible was allowed to remain in the furnace for an additional 55 min. Subsequently, the crucible was allowed to cool to ambient temperature within a desiccator. The constant mass (m_2_) was determined through a series of successive 1-h ignitions, each followed by a cooling period and subsequent weighing. The LOI was computed as follows:

(3) $$LOI=\frac{m_{1}-m_{2}}{m_{1}}\times 100\;[\%]$$

where:

- m_1_—mass of sample before heating, g;
- m_2_—the constant mass of examined sample, g.

#### 2.1.4. Particle Size Distributions (PSDs)

**Figure 1:**
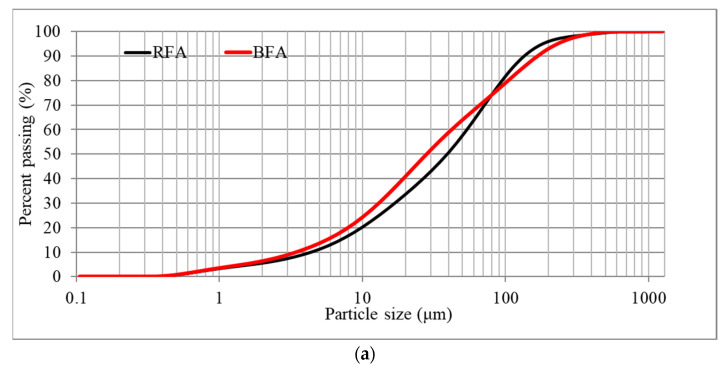

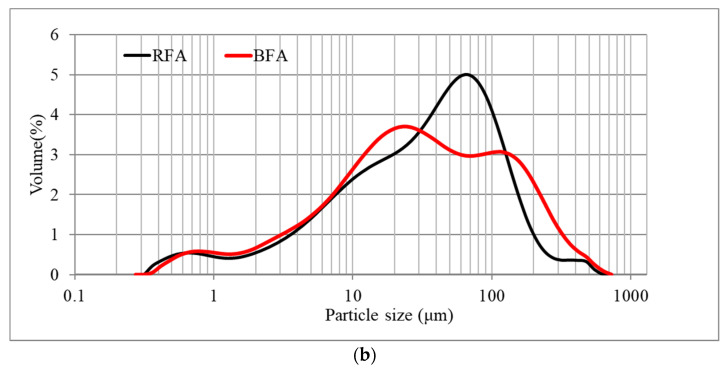
Raw materials’ particle size distribution. (**a**) Cumulative particle size distribution curves, (**b**) differential particle size distribution curves.

The Malvern Mastersizer 2000 laser diffraction instrument, along with its Hydro 2000 S dispersion unit, was employed for the fly ashes particle size analysis depicted in Figure 1. The PSDs of the fly ashes were assessed utilizing a wet dispersion laser diffraction technique, with a detection range of particle diameters from 0.02 µm to 2000 µm. This apparatus applies Mie theory and static light scattering principles to ascertain the dimensions of particles within a sample. In essence, smaller particles scatter light at broader angles, while larger particles scatter light at more acute angles. The scattering pattern generated by the sample powder is documented, allowing the calculation of particle size distribution through Mie scattering theory.

#### 2.1.5. SEM Image Analysis

A Thermal Field Emission Scanning Electron Microscope (FE-SEM, Hitachi, Tokyo, Japan) with a Bruker EDS analyzer was utilized for the morphological assessment of fly ashes and mortar samples. Fly ash samples were deposited on carbon tape to form a thin powder layer. Subsequently, they were coated with a thin layer of gold. Imaging was conducted at various magnifications under high vacuum conditions with an accelerating voltage of 5 kV. Mortar specimens were extracted from beam samples measuring 4 cm × 4 cm × 16 cm. One-centimeter thick samples were sectioned from the beams, dried, and prepared for SEM analysis by applying a thin gold film under vacuum conditions.

### 2.2. Alkali Activated Mortars

The investigation was conducted on alkali-activated materials (AAM) formulated in accordance with the following principles:

- Aluminosilicate precursors: mix of bituminous coal combustion fly ash (RFA) and wooden biomass combustion fly ash (BFA) from Polish Power Heat and Power Plant Siekierki, Warsaw, Poland;
- Alkaline activators: 30% sodium hydroxide solution—7.5 M (PCC, Brzeg Dolny), sodium silicate with SiO_2_/Na_2_O ratio between 1.9 and 2.1 (R150, Z.Ch. Rudniki S.A., Rudniki k. Częstochowy, Poland);
- Phosphate compound: Tetraphosphorus decaoxide—P_4_O_10_ (Chempur, Piekary Śląskie, Poland);
- Fine aggregate: Standard Sand according to EN 196-1 [32].

All formulations of geopolymer mortar were designed and developed in accordance with a modified methodology derived from EN 196-1, as outlined below:

- Introduce geopolymerization precursor into the mixer and maintain low rpm (140 ± 5 rpm);
- Introduce sand uniformly for the initial 30 s of mixing;
- Incorporate alkaline activator solution steadily for the subsequent 30 s;
- Shift the mixer to high rpm (285 ± 10 rpm) and continue mixing for an additional 30 s.

Prepared samples were demolded after 24 h, cured firstly in an oven at 65 °C for 48 h and secondly kept in laboratory conditions (21 ± 1 °C, 50% relative humidity) until testing. The curing procedure was established based on a literature review.

Geopolymer mortars were prepared by mixing RFA and BFA in ratio 7:3 (see Table 1). This proportion allowed to obtain fly ash mix comparable in chemical composition to fly ash taken from co-firing of wooden biomass and coal also produced at the Polish Heat and Power Plant Siekierki. To obtain M5 class mortar, a binder/sand ratio equal to 1.5 was used based on EN 998-2 [33], PN-B-10104 [34] standards and research experience. Fly ashes are activated with an aqueous hydroxide solution with a NaOH/Na_2_SiO_3_ ratio equal to 2.7. The quantity of activator has been selected so as to obtain a consistency of approximate 15 cm in flow measurement (EN 1015-3 [35]). Tetraphosphorus decaoxide is added to fly ashes before its alkali-activation with quantities ranging from 1% to 5% of the total mass of the aluminosilicate precursors mass. The maximum level of tetraphosphorus decaoxide addition is based on maximum content of phosphate compound in used biomass fly ash during it chemical compound testing through 2 years (2021–2022).

The nomenclature of the specimens presented in Table 1 is based upon established designations “MFA-P#-”, where “MFA” is the mix of BFA and RFA, “P#” is a symbol representing the level of P_4_O_10_ in mix. Flexural and compression tests, porosity measurement with micro computed tomography (μCT), Scanning Electron Microscopy (SEM), Fourier Transform Infrared Spectroscopy (FTIR) were used to examine properties of obtained alkali-activated materials.

#### 2.2.1. Compressive and Flexural Strengths

Evaluations of compressive and flexural strength were carried out in accordance with PN-EN 196-1 standards, utilizing beam specimens measuring 4 cm × 4 cm × 16 cm and a hydraulic press fitted with appropriate attachments. The assessments were conducted at 7 and 28 days following the preparation of the mortar. The evaluations utilized a control system alongside an Instron hydraulic press (Instron, Norwood, MA, USA) operating at a compressive loading rate of 0.33 MPa/min (sensitivity 100 kN), and a flexural loading rate of 0.017 MPa/min (sensitivity 5 kN). The results were derived as the average of three samples for flexural strength and six samples for compressive strength.

#### 2.2.2. Porosity

Micro computed tomography (μCT) was performed on selected samples on an XRADIA XCT-400 tomograph (Xradia, Concord, CA, USA). The measurements were carried out at a lamp voltage of 150 kV and a current of 60 μA. During the μCT measurement, 900 X-rays images with exposure time 8 s and resolution of 25 μm were obtained. Each of them were created for a given angular position of the 4 cm high sample in the range of 0–180 degrees. Based on the obtained images, cross-sections of the sample were reconstructed using the modified Feldkamp algorithm. From the reconstruction process, over 900 virtual X-Y cross-sections were obtained, covering a two-dimensional sample area.

#### 2.2.3. Fourier Transform Infrared Spectroscopy (FTIR)

The utilization of FTIR spectroscopy was employed to ascertain the mineral composition and phase transitions predicated on the examination of distinct molecular configurations and the identification of structural formations engendered during the polycondensation of geopolymers. The specimens intended for FTIR analysis were prepared through the KBr Pellet Method. This methodology capitalizes on the characteristic of potassium bromide (KBr), an alkali halide, which acquires a plastic state upon the application of pressure, thereby facilitating the creation of a transparent sheet within the infrared spectrum. The procedural steps for the preparation of the pellet were as follows:

- Approximately 0.1 to 1.0% of the sample was amalgamated with 200 to 250 mg of finely ground KBr powder;
- The resultant mixture was meticulously pulverized and subsequently placed into a die designated for pellet formation;
- A compressive force of approximately 8 tons was exerted under a vacuum of several mm Hg for a duration of several minutes to yield transparent pellets.

Prior to conducting the measurements, a background spectrum was recorded utilizing an empty pellet holder inserted into the sample chamber. Conducting the background measurement with a KBr pellet devoid of any sample facilitated the correction for infrared light scattering losses within the pellet as well as for moisture adsorbed onto the KBr.

## 3. Results and Discussion

### 3.1. Fly Ashes

#### 3.1.1. Chemical Compositions, Mineral Phase Characterizations, and Physical Characteristics

RFA demonstrates the requisite chemical properties as stipulated in EN 450-1 and ASTM C618 [36], which corresponds to Class F fly ash (approximately 84.34% of the RFA composed SiO_2_, Al_2_O_3_ and Fe_2_O_3_). The low concentration of calcium oxide (3.51%) and average loss of ignition (LOI = 5.50%) adhere to the specifications established in the aforementioned standards. The predominant crystalline phases observed in the RFA were identified as mullite and quartz. It is coherent with other authors analyses [37].

Generally, the aluminosilicate compounds present in fly ashes obtained from wooden biomass exhibit lower concentrations than those found in coal fly ashes [38,39]. The silica, aluminum, and iron content in biomass fly ash (BFA) is less when set against the residual fly ash (RFA) samples, with SiO_2_, Al_2_O_3_, and Fe_2_O_3_ combining to 56.16%. A corresponding reduction was noted in the amorphous content, which stands at 38.2%. It is plausible that these compositional discrepancies arise from the biomass fraction incorporated during firing, as wooden biomass fly ashes are predominantly crystalline, a phenomenon corroborated by prior research investigations [40,41]. The primary oxide concentrations in BFA fall below the anticipated range for coal fly ash, notably exhibiting elevated levels of potassium and phosphorus (7.96% and 2.46%, respectively). The BFA presents a substantially higher level of loss on ignition (LOI) at 7.88%. Nevertheless, this specific LOI measurement adheres to the EN 450-1 standard, thereby categorizing BFA as a Class C fly ash.

Minerals containing potassium associated with biomass fuel have been identified via X-ray diffraction (XRD) in BFA, manifesting as arcanite, leucite, and alunite. Furthermore, BFA demonstrates a greater enrichment in magnesium and manganese oxides in comparison to RFA. These two elements are recognized as vital nutrients for wooden biomass, as delineated by Szostek et al. [42]. The calcium content in BFA is similarly heightened relative to RFA, with XRD analysis revealing the presence of calcite and a minor quantity of portlandite as the crystalline components containing calcium.

#### 3.1.2. PSDs

Table 2 and Figure 1 illustrate the diameter characteristics and particle size distributions (PSDs) for all examined ashes. The mean particle sizes for the coal and biomass fly ashes were found to be remarkably comparable, with values of 54.32 and 58.50 μm, respectively. The marginally greater particle size of the biomass fly ash (BFA) is likely attributable to factors such as incomplete combustion, reduced firing temperatures, the utilization of wooden biomass, or a combination of the aforementioned elements [43,44,45]. An analysis of the particle size distribution graphs (Figure 1) suggests that both raw fly ash (RFA) and BFA samples exhibit a homogenized particle composition. In the case of BFA, larger particles approaching a dimension of 150 µm can be distinctly observed.

#### 3.1.3. SEM Analysis

**Figure 2:**
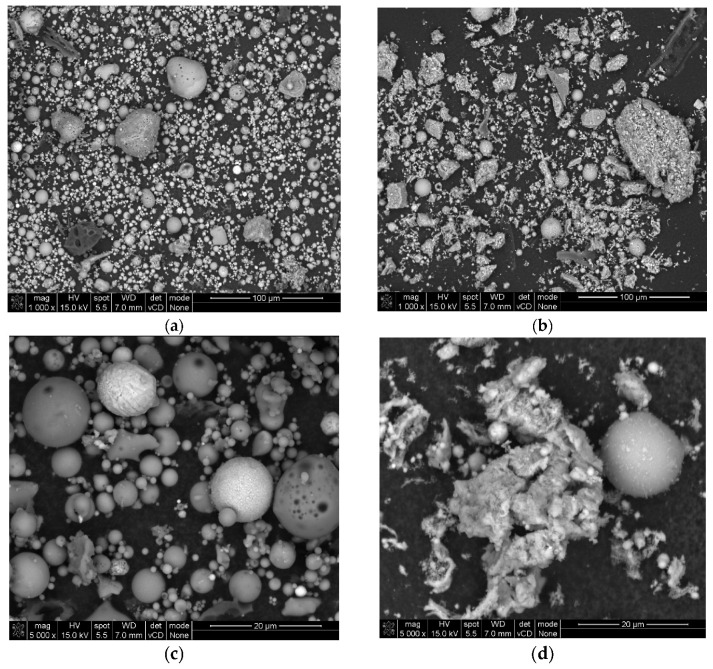
SEM pictures in 100-µm and 20-µm scale of fly ashes: (**a**,**c**) RFA, (**b**,**d**) BFA.

**Figure 3:**
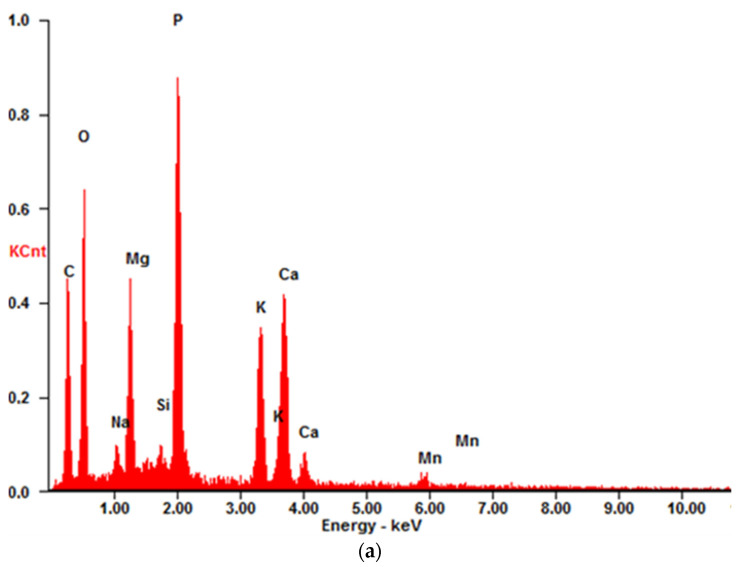

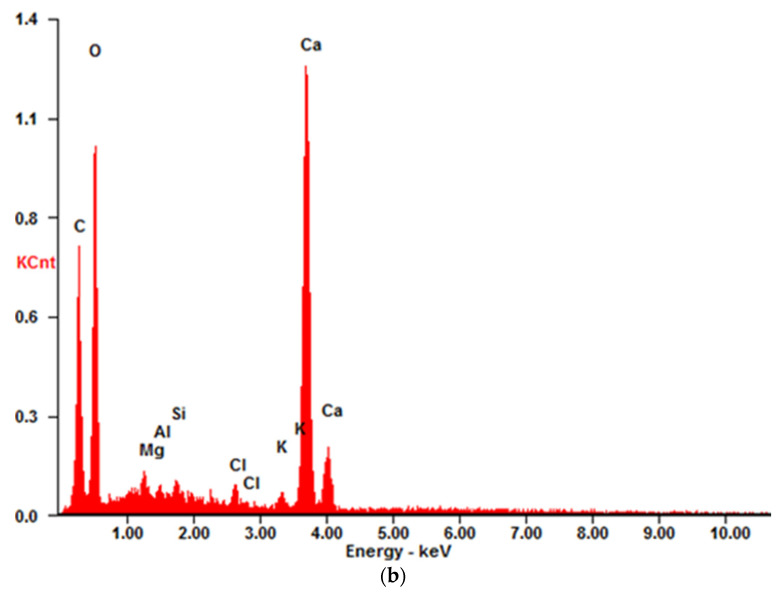
Examples of Espirit Spectrum Analysis for Bruker EDS: (**a**) BFA, point with higher levels of phosphate, calcium, and potassium compounds, (**b**) RFA, point analyzed as calcite.

The micrographs captured at a scale of 100 µm distinctly illustrated the spherical morphology of the particles present in RFA fly ash (Figure 2a). Energy Dispersive Spectroscopy (EDS) analysis substantiated that, in the case of RFA, these particles predominantly comprised silica and aluminum compounds characteristic of glassy aluminosilicates [46]. In contrast, BFA exhibited a heterogeneous composition of both spherical and coarse particles, which are indicative of silica, phosphate, calcium, and potassium compounds (Figure 3a).

The RFA cenosphere particles were predominantly identified as non-porous and devoid of any deformities (Figure 2c). In the case of BFA, particles characterized by elevated calcium concentrations were noted. Subsequent analysis utilizing the Bruker Espirit Spectrum facilitated the characterization of these particles as calcite (Figure 3b). Additionally, elongated, fibrous particles exhibiting intact cells of a woody morphology were observed. This observation further suggests that the raw wooden biomass used in the production of BFA was not subjected to complete combustion.

### 3.2. Fly Ash Mortars

#### 3.2.1. Flexural and Compressive Strengths

**Figure 4:**
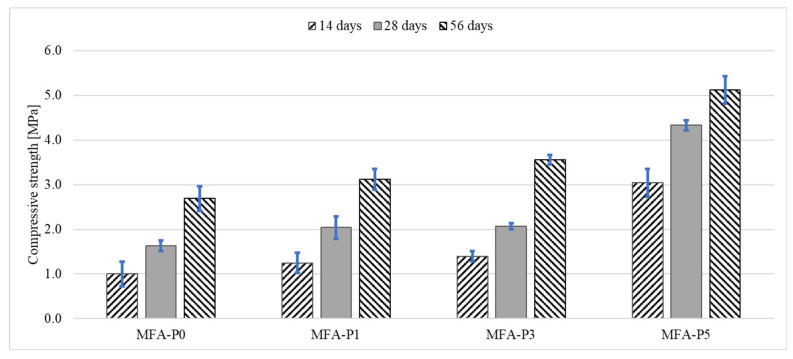
Flexural strength development of mortars with different level of P_4_O_10_.

The incorporation of phosphoric oxide into geopolymers has been documented to yield a significant improvement in the flexural strength of the composite material produced (Figure 4). The maximal flexural strength values were recorded in the specimens formulated with a phosphoric oxide concentration of 5%. As demonstrated in the accompanying figure, it is essential to note that the results associated with the 5% phosphoric acid solution distinctly deviate from the other findings. The mortars containing 1% and 3% P_4_O_10_, despite exhibiting a resemblance to the control mortar in the graphical depiction, also reveal a significant enhancement in strength. Following 14 and 28 days, the strength of these mortars is approximately 25% superior to that of the reference mortar. After a period of 14 days, the increase in shear strength for each of the mortars containing P_4_O_10_ is pronounced, with the mortar incorporating 5% P_4_O_10_ displaying a flexural strength equal to 3.04 MPa. Consequently, it can be inferred that the predominant increase in strength transpires during the initial weeks of sample curing, as indicated by the reduced disparities observed after 28 and 56 days, respectively, with the highest result for MFA-P5 obtaining 5.12 MPa.

**Figure 5:**
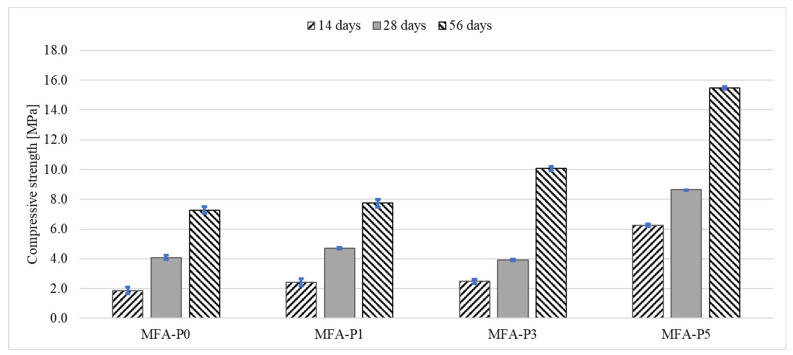
Compressive strength development of mortars with different level of P_4_O_10_.

Across all examined specimens, a uniform augmentation in compressive strength is discerned as the curing duration extends from 14 days to 56 days (Figure 5). This phenomenon is characteristically observed in geopolymer materials, wherein ongoing geopolymerization facilitates strength enhancement over temporal progression [47]. The escalating compressive strength intimates that the materials are experiencing additional chemical bonding and densification, thereby reinforcing their structural integrity.

The phosphate oxide concentration within the specimens demonstrates a distinct positive correlation with compressive strength across all temporal intervals. The reference specimen (MFA-P0) manifests the lowest compressive strength at each time point assessed. The strength escalates from approximately 2 MPa at 14 days to nearly 8 MPa at 56 days. The incorporation of 1% phosphate oxide engenders a significant improvement in strength relative to MFA-P0, indicating that even minimal concentrations of P_4_O_10_ can enhance material performance. A 3% P_4_O_10_ concentration further amplifies compressive strength, suggesting a more pronounced influence of phosphate oxide on the structural properties of the material. Specimens augmented with a 5% addition of P_4_O_10_ exhibit the utmost compressive strength throughout all assessed time intervals. Beginning at approximately 6 MPa at 14 days, it culminates at around 16 MPa at 56 days.

The effect of phosphate compounds on compressive and flexural strengths of alkali-activated mortars are also observed by other authors [48,49,50,51]. In alkali-activated mortars made from phosphate mine tailings, the addition of slag enhances the formation of N-A-S-H gels, which are crucial for improving compressive strength. The compressive strength increases with the addition of slag up to 40%, after which it plateaus, indicating that phosphate, in conjunction with slag, contributes to the strength development by facilitating gel formation [48]. In contrast, the addition of fly ash, which is less reactive than phosphorous slag, reduced the early age strength of the alkali-activated materials [49,51]. It is observed that the balance between phosphate and other components like slag and fly ash is crucial. While slag contributes to denser materials, fly ash tends to create more porous structures, which can affect the mechanical properties [50]. Moreover, the chemical composition of the materials, including the presence of elements like aluminum and calcium, also plays a critical role in the formation and properties of the final materials, influencing both compressive and flexural strength [50]. Therefore, the influence of phosphate on compressive and flexural strength is multifaceted, depending on its interaction with other materials and the specific composition and processing conditions of the alkali-activated systems. In this study, the influence of phosphate one mechanical properties of fly ash geopolymer mortars is analyzed through porosity, FT-IR, and XRD analysis.

#### 3.2.2. Porosity

**Figure 6:**
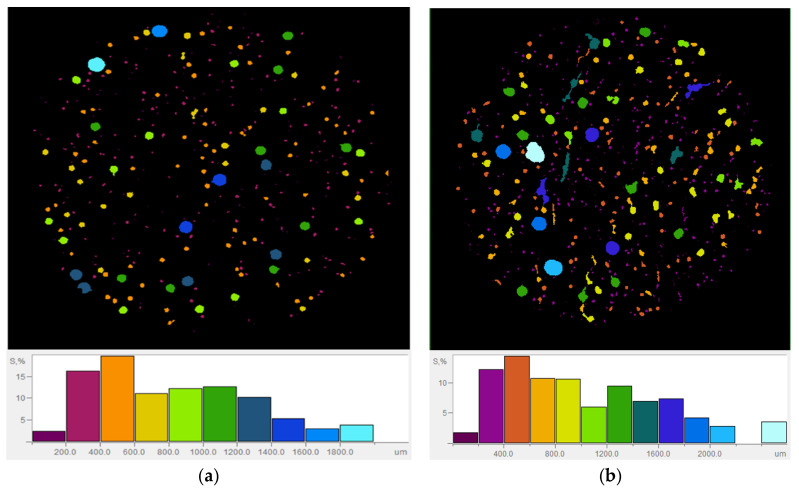
The cross sections and pores structure from the uCT test: (**a**) MPA-P0, (**b**) MPA-P5.

In order to enhance the comprehension of pore distribution in the formulated mortars, samples MFA-P0 and MFA-P5 were selected for analysis utilizing the X-ray microtomography (μCT) technique (Figure 6a,b). The reference mortar exhibited a porosity that was at least twofold lower than that of the sample containing a 5% addition of P_4_O_10_ (specifically 4.26% and 9.98%, respectively). The MFA-P0 sample demonstrated a mean pore size that was reduced by nearly 12% (measured at 450 µm and 510 µm, respectively). Within the MFA-P5 mortar, a substantial quantity of closed spherical pores was found to co-exist with microcracks. This degradation of structure may be attributed to the formation of amorphous calcium phosphate [52].

#### 3.2.3. FTIR

**Figure 7:**
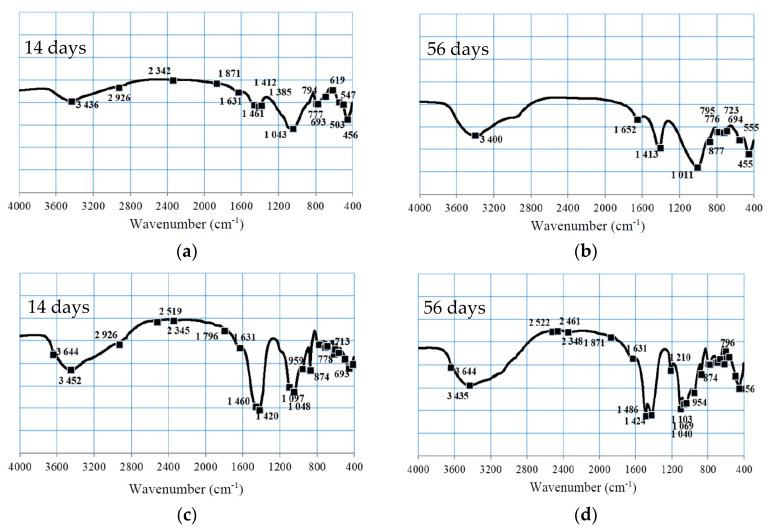
FT-IR spectra of prepared mortars after 14 and 56 days of curing: (**a**,**b**) MFA-P0, (**c**,**d**) MFA-P5.

The spectral bands of significant interest in this investigation pertain to the stretching and bending vibrational modes of Si-O-Si(Al) bonds situated in a tetrahedral configuration, along with those associated with the stretching vibrations of carbonate functional groups [53]. Notable alterations between the samples after 14 days and 56 days of curing are predominantly evident in the Si-O-(Si,Al) (1300–900 cm^−1^), C-O (1460–1420 cm^−1^), and P=O (1275–990 cm^−1^) spectral regions (Figure 7). In the context of geopolymers, the asymmetrical stretching vibrations of the Si-O-T bond (where T denotes either Si or Al) are typically identified within the 1000–1100 cm^−1^ spectral range [54].

**Figure 8:**
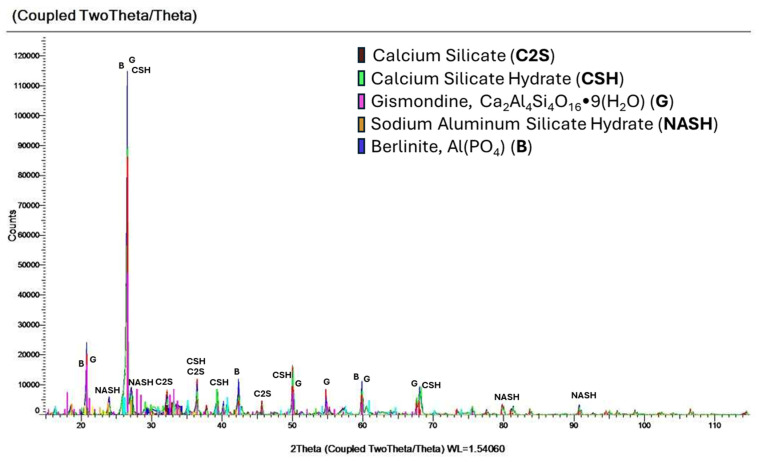
XRD spectra of MFA-P5 mortar after 56 days of curing.

In the MFA-P0 and MFA-P5 samples, the peaks corresponding to the Si-O-T bond were detected at wavenumbers ranging from 1043 to 1048 cm^−1^ following 14 days of curing, indicating the potential formation of Si-O-Si bonds within the resultant C-S-H gel matrix [55]. Nevertheless, the pronounced peak observed in this spectral region for MFA-P5 may imply the presence of Si-O-Si bonds. As the MFA-P5 samples undergo aging, the asymmetrical stretching vibration of Si-O-Si transitions to higher wavenumbers (approaching 1100 cm^−1^), a characteristic indicative of C-S-H and N-A-S-H gel structures [56]. The occurrence of C-S-H and N-A-S-H gel was confirmed by XRD analysis (Figure 8). The intensities of the bands ascribed to OH group vibrations within the 3851–3400 cm^−1^ range were also markedly more pronounced in the MFA-P5 mortars. This is probably connected with presence of N-A-S-H, C-S-H and microcrystalline zeolite phase—gismondine. Both N-A-S-H and C-S-H can potentially enhance mechanical properties as curing duration increases [56]. It is plausible that the presence of phosphate is facilitating the dissolution of amorphous fly ash phases, thereby augmenting the geopolymerization process. The XRD analysis of MFA-P5 samples indicates also the formation of a new crystalline phase berlinite, which acts as a filler and reinforces the structure and therefore the compressive strength of the examined mortar [57].

The broad peak observed in the MFA-P5 samples after 56 days within the 1210–874 cm^−1^ range suggests that their internal structural integrity may have undergone a more significant degree of depolymerization, attributed to the role of Ca ions as network modifiers that promote the dissolution of aluminum [58]. These alterations may also be correlated with the incorporation of both Na and Ca ions into the phosphate network structures.

In the MFA-P5 samples, the spectral bands associated with the stretching vibrations of C-O and bicarbonates (1486–1463 cm^−1^) exhibited strong and broad peaks. This phenomenon may result from the formation of sodium carbonate (Na_2_CO_3_) due to the reaction between NaOH and atmospheric CO_2_ [59,60]. Elevated concentrations of Na_2_CO_3_ within geopolymer mortars may significantly influence their mechanical and durability characteristics owing to its solubility in water.

#### 3.2.4. SEM

**Figure 9:**
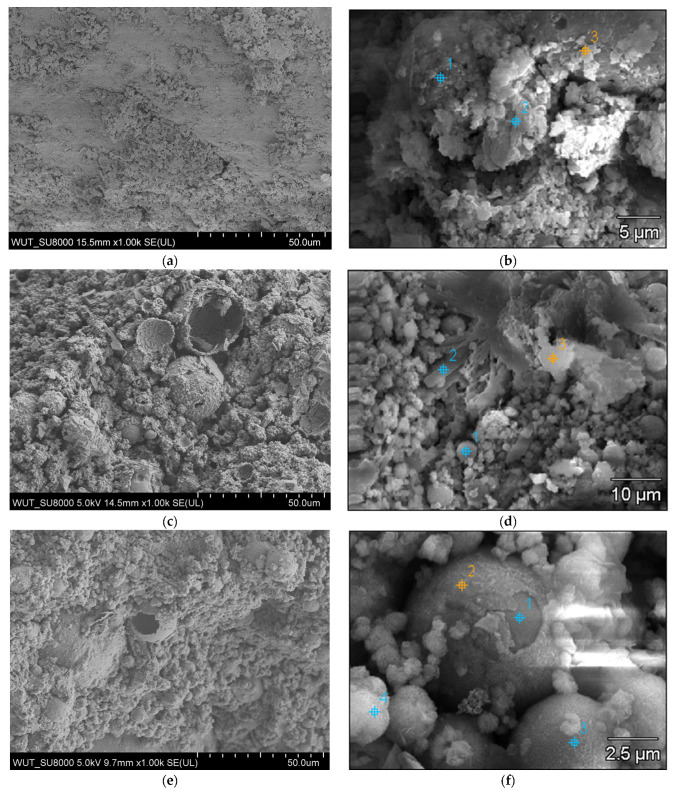
SEM pictures with EDS mapping of mortars with different level of phosphate compound (**a**,**b**) MFA-P0, (**c**,**d**) MFA-P3, and (**e**,**f**) MFA-P5.

**Figure 10:**
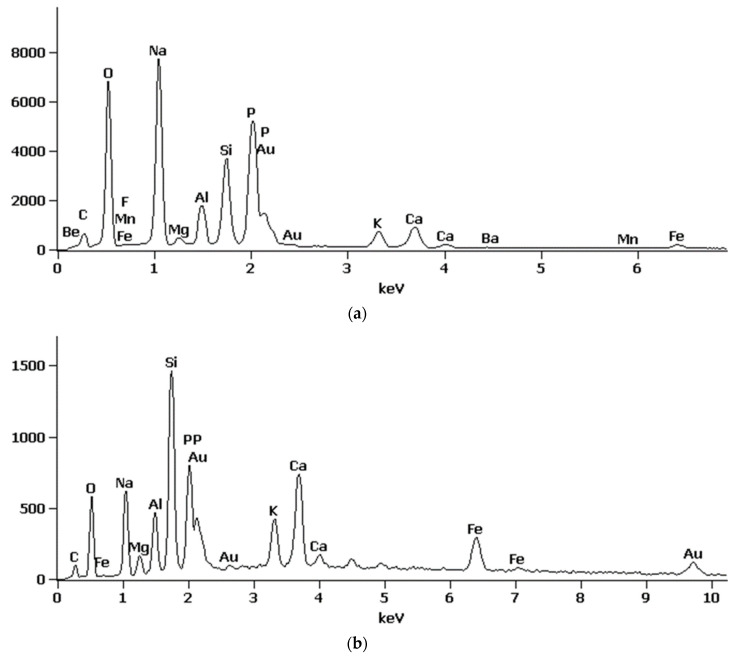
Examples of Espirit Spectrum Analysis for Bruker EDS: (**a**) BFA, point 2 from Figure 9d, (**b**) BFA, point 4 from Figure 9f.

SEM images of MPA-P0, MPA-P3 and MPA-P5 are presented in Figure 9, respectively. It can be seen that the surface morphology of MPA-P0 showed the relatively denser microscale structures versus other samples (Figure 9a,b). In the case of the MFA-P3 and MFA-P5 samples, the exothermic reaction of phosphate and calcium presence could provide extra nucleation sites for precipitation of dissolved ions and trigger rapid hardening, leaving more coarse gel structure [61]. The coarse gel morphology identified in SEM images exhibits resemblance to the particulate frameworks of amorphous calcium and sodium phosphate [62]. This correlation was substantiated through EDS analysis conducted at points 2 and 4 (as illustrated in Figure 10a and Figure 10b, respectively), which were characterized by the predominant elements: phosphorus, sodium, calcium, and oxygen.

At the same time, the microstructures of the MFA-P3 and MFA-P5 samples (Figure 9d and Figure 9f, respectively) presented reacted amorphous microspheres, and some partially reacted fly ash spheres in the entire matrix. In specific sections of those samples, some small shaped pores were developed in the matrix probably after the dissolution of fly ash particles [63].

## 4. Conclusions

The influences of phosphate oxide addition from 1% to 5% of the total mass of the aluminosilicate precursors mass on the properties of fly ash geopolymers were analyzed. Based on the results, key findings can be summarized as follows:

- The concentration of P_4_O_10_ present within the samples exhibits a positive relationship with both flexural and compressive strength across all assessed temporal intervals (7, 28, and 56 days).
- The incorporation of P_4_O_10_ leads to a marked increase in porosity of geopolymer mortars.
- The addition of 5% P_4_O_10_ facilitated the emergence of a new crystalline phase, identified as berlinite.

## Figures and Tables

**Table 1:** Mortar mix designs.

Mix Type	RFA (g)	BFA (g)	Alkaline Activator (g)	Sand (g)	P_4_O_10_ (g)	Water (g)
MFA-P0	1575	675	1464	3375	0	0
MFA-P1	1575	675	1464	3375	22.5	0
MFA-P3	1575	675	1464	3375	67.5	0
MFA-P5	1575	675	1464	3375	112.5	0

**Table 2:** Fly ashes characteristics.

Chemical Composition (%) and Physical Characteristic	RFA	BFA
SiO_2_	51.73	50.98
Al_2_O_3_	26.20	3.38
Fe_2_O_3_	6.41	1.80
MnO	0.09	0.68
MgO	2.88	3.98
CaO	3.51	17.40
Na_2_O	1.18	0.54
K_2_O	2.92	7.96
TiO_2_	0.20	1.05
P_2_O_5_	0.34	2.43
Loss On Ignition, LOI (%)	5.50	7.88
Total of XRF	100.18	97.28
∑(SiO_2_ + Al_2_O_3_ + Fe_2_O_3_)	84.34	56.16
Real density (g/cm^3^)	2.200	2.352
Mean particle size (μm)	54.32	58.50
**Mineral Composition (%)**		
Quartz	13.4	38.2
Mullite	14.8	–
Calcite	–	8.9
Portlandite	–	1.4
Hematite	–	1.0
Orthoclase	–	–
Microcline	–	–
Alunite	–	2.4
Anhydrite	–	0.3
Arcanite	–	8.2
Leucite	–	1.9
Archerite	–	-
Amor	67.8	38.2

## Data Availability

The original contributions presented in the study are included in the article, further inquiries can be directed to the corresponding author.
